# Coordinated physiological and molecular reprogramming by brassinosteroids improves soybean tolerance to combined salt and drought stress

**DOI:** 10.1080/15592324.2026.2616539

**Published:** 2026-01-20

**Authors:** Tanveer Alam Khan, Taiba Saeed, Lam Son Phan Tran, Mayank Anand Gururani

**Affiliations:** a Department of Biology, College of Science, United Arab Emirates University, Al Ain, United Arab Emirates; b Department of Biosciences, Integral University, Lucknow, Uttar Pradesh, India; c Department of Plant and Soil Science, Institute of Genomics for Crop Abiotic Stress Tolerance, Texas Tech University, Lubbock, TX, USA

**Keywords:** Brassinosteroids, soybean, abiotic stress, photosynthesis, antioxidant, proline metabolism

## Abstract

This study investigates how brassinosteroids (BRs) enhance stress tolerance in soybean under combined salt and drought stress by examining growth, chlorophyll content, photosynthesis, and reactive oxygen species (ROS) homeostasis. Salt and drought stress significantly reduced soybean growth and photosynthetic performance, as reflected by lower SPAD chlorophyll values and decreased photosystem II (PSII) efficiency. In contrast, BR (24-epibrassinolide, EBL) significantly improved growth parameters and spectral indices, indicating a healthier pigment status and improved canopy function. EBL-treated plants also exhibited enhanced PSII performance, as indicated by increased Fv/Fm and a higher performance index (PI). Furthermore, BRs modulated ROS levels and promoted cellular homeostasis by elevating the activities of antioxidant enzymes such as APX, CAT, and POX, thereby mitigating oxidative damage. Consistently, expression of key stress-responsive genes (*GmCAT, GmSOD*, and *GmP5CS*) was strongly induced under combined salt, drought, and EBL treatment, highlighting the synergistic role of EBL in transcriptional activation under combined stress. EBL treatment increased the proline content and the activities of ProDH and P5CS, supporting proline-mediated osmoprotection, while BR-treated plants exhibited reduced malondialdehyde (MDA) accumulation and electrolyte leakage (EL), indicating lower lipid peroxidation and better membrane integrity under stress. Overall, this study demonstrates that EBL enhances soybean resilience to combined salt and drought stress by improving growth, photosynthetic efficiency, antioxidant defense, osmotic adjustment, and membrane stability.

## Introduction

Drought and salt stress are two significant environmental challenges facing plants today, particularly as climate change exacerbates their frequency and severity.^1^ Both conditions severely affect plant growth, development, and overall productivity, leading to substantial agricultural losses and threatening food security.^2^^,^^3^ These constraints are especially critical in arid and semi-arid regions, where limited and irregular rainfall, high evaporative demand, and progressive soil degradation increasingly restrict the range of crops that can be cultivated. Soybean, a major source of plant protein and vegetable oil, is being expanded into such marginal environments to meet rising global demand, but its productivity is highly sensitive to water limitation and salinity. Drought stress arises when soil water availability is insufficient to meet plant physiological demands, often owing to prolonged low rainfall, high temperatures, and increased evaporation rates.^4^ In response, plants close their stomata to limit transpirational water loss, but this also restricts CO₂ uptake, thereby constraining photosynthesis and growth.^5^^,^^6^ To maintain cell turgor, they adjust osmotically by accumulating compatible solutes such as sugars and amino acids, which help stabilize their cellular functions under reduced water availability.^7^ These adjustments are accompanied by changes in root architecture, including deeper or more extensive root systems that improve access to remaining soil moisture, and by alterations in hormone signaling networks that modulate growth, senescence, and stress responses. Moreover, salinity stress likewise poses a major abiotic constraint to global agriculture by reducing crop productivity and compromising sustainability.^8^ High salinity levels impair plant morphology and biochemical processes, inhibiting seed germination, growth, development, and yield.^9^^,^^10^ Salinity adversely affects the photosynthetic apparatus by reducing chlorophyll and carotenoid content, distorting chloroplast structure, impairing PSII function, and lowering stomatal conductance, collectively disrupting photosynthesis, transpiration, and gas exchange.^3^^,^^11^^,^^12^ In saline soils, reduced soil and leaf water potential interferes with plant water relations, while excessive Na⁺ and Cl⁻ accumulation and reduced K⁺ and Ca²⁺ uptake lead to ionic imbalance and disturbed mineral nutrition.^13^^,^^14^ Because drought and salinity frequently co-occur in arid and semi-arid regions, plants are often challenged simultaneously by osmotic and ionic stresses, resulting in more severe limitations to growth and productivity than either stress alone.

Brassinosteroids (BRs) are fundamental plant hormones that regulate a wide range of physiological and developmental processes, significantly contributing to plant growth, productivity, and resilience against environmental stressors.^15^ These hormones are now recognized as key regulators of cellular activities such as cell elongation, division, vascular tissue differentiation, and reproductive development.^16^ BRs influence multiple molecular pathways, including those involved in growth regulation, photosynthesis, and stress responses.^17^^,^^18^ They improve photosynthetic performance by enhancing chlorophyll synthesis and promoting carbon fixation efficiency.^19^^,^^20^ In addition to their roles in growth promotion, BRs are critical for enhancing plant tolerance to various abiotic stresses. They have been shown to improve resilience to chilling stress,^21^ mitigate the toxic effects of heavy metals,^22^ and help plants endure high-temperature conditions.^23^ BRs achieve this by activating antioxidant defense mechanisms, which reduce oxidative stress by minimizing reactive oxygen species (ROS) accumulation.^20^^,^^24^ Moreover, these hormones regulate ion balance and osmotic adjustment, which are crucial for maintaining water homeostasis during stress.^25^ At the molecular level, BR signaling interacts with other hormone pathways, such as abscisic acid and auxin, and modulates the expression of stress-responsive genes involved in ROS detoxification, osmolyte biosynthesis, and membrane transport. Among the various BR analogs, 24-epibrassinolide (EBL) stands out for its protective effects against a wide range of environmental stresses, including heat, drought, low temperatures, heavy metal exposure, and salinity.^3^^,^^23^^,^^26-30^ Accordingly, this study aims to advance our understanding of BR-mediated stress tolerance by evaluating the capacity of EBL to mitigate the combined effects of salt and drought stress in soybean, focusing on key physiological parameters such as growth dynamics, chlorophyll indices, photosynthetic efficiency, ROS homeostasis, and gene expression.

## Materials and methods

### Biological materials

Authenticated soybean seeds supplied by Modern Agri. & IRRI. EST (Al Ain, UAE) were inspected for uniformity and physiological viability prior to experimentation. Seeds belonging to batches that met the established viability threshold underwent surface sterilization using a 1% sodium hypochlorite solution for 10 min. They were then rinsed thoroughly three times with double-distilled water (DDW) to eliminate residual sterilant and ensure aseptic conditions before sowing.

### Preparation for EBL

24-Epibrassinolide (EBL) was obtained from Sigma–Aldrich, USA. A stock solution was initially prepared at a concentration of 10⁻⁴ M and then diluted to 10⁻⁸ M to meet the experimental requirements, following the specified methodology by Khan et al.^29^

### Experimental design with treatment pattern

Sterilized soybean seeds were sown into forty uniformly sized plastic pots (10 inches in diameter), each filled with a 1:1 mixture of sand and peat soil. The pots were then maintained in a greenhouse under controlled environmental conditions. They were then categorized into eight groups, each consisting of five pots, to serve as replicates for different treatments.

**Table:** 

Set	Treatment description
Set I (control)	No stress or EBL; foliage sprayed with deionized water at 26, 27, 28, 29, and 30 d of growth
Set II	Foliage sprayed with 10^–8^ M of EBL at 26, 27, 28, 29, and 30 d of growth
Set III	Salt stress (200 mM) was applied at 15, 16, 17, 18, and 19 d of growth
Set IV	Drought stress induced using 20% PEG through the soil at 15, 16, 17, 18, and 19 d of growth
Set V	Combination of set III (salt stress) and set IV (drought stress)
Set VI	Combination of set II (EBL spray) and set III (salt stress)
Set VII	Combination of set II (EBL spray) and set IV (drought stress)
Set VIII	Combination of set II (EBL spray), set III (salt stress), and set IV (drought stress)

At the 40-d growth stage, the plants were harvested to assess their physiological features, biochemical parameters, and growth biomarkers. Each assay was repeated 5×, with 15 plants utilized per treatment (three plants per pot).

### Assessment of growth characteristics

From each pot, one plant was randomly selected and thoroughly rinsed under running water to eliminate all the sand particles. Excess surface water was removed by gently blotting the specimens with absorbent sheets. The root and shoot lengths were quantified using a meter scale. Fresh biomass was determined using an electronic balance, after which the samples were placed in a drying oven at 70 °C for 3–4 d to achieve constant weight. The dried samples were then reweighed on the same balance to obtain dry biomass values.^31^

### Leaf spectrometer measurements

Leaf spectral traits were measured on the fully expanded third leaf from the top of each plant using a calibrated Spectavue CI-710S Leaf Spectrometer (CID-BioScience, USA). The spectral data were analyzed to estimate plant tolerance and compute stress-related indices, including SPAD, CRI1, WBI, NPCI, ARI1, NPQI, PRI1, CCI, the greenness index, and ZMI, as described in our previous paper.^31^

### Chlorophyll-a fluorescence measurements

Chl-a fluorescence measurements were assessed following the procedure described in previous studies AlNeyadi et al.^11^

By making a few minor modifications, the method outlined by Usuda^32^ was applied to assess Rubisco activity.

### Estimation of antioxidant enzyme activities

The activities of catalase (CAT), peroxidase (POX), and superoxide dismutase (SOD) in leaf tissues were analyzed as indicators of antioxidant capacity. The samples were homogenized in an extraction buffer containing PVP, Triton X-100, PMSF, and EDTA, then centrifuged at 12,000 × *g* for 20 min at 4 °C, with the supernatant retained for assays. CAT activity was determined using the Aebi^33^ method by monitoring H₂O₂ decomposition at 240 nm. POX activity was evaluated following Sánchez et al.^34^ by recording guaiacol oxidation at 436 nm. SOD activity was measured according to Beauchamp and Fridovich,^35^ with absorbance at 560 nm quantified as outlined in our previous work.^31^

### Analysis of proline metabolism (proline content, ProDH, and P5CS activities)

A newly collected leaf sample (0.5 g) was ground in 3% sulfosalicylic acid. The resulting homogenate was centrifuged at 10,000 × *g* for 10 min to separate the supernatant. A fresh reaction mixture was then prepared in a new tube according to the protocol outlined by Bates et al.^36^ The mixture was heated in a water bath at 100 °C for 1 h and then quickly cooled by transferring the tubes to an ice bath. After cooling, toluene was added, and vigorously mixed to extract the chromophore layer. The optical density of the resulting red-colored chromophore was measured at 520 nm.

The enzymatic activities of pyrroline-5-carboxylate synthase (P5CS) and proline dehydrogenase (ProDH) were evaluated following the procedure outlined by Yusuf et al.^37^

### Measurement of stress biomarkers (H_2_O_2_ content, electrolyte leakage, and lipid peroxidation)

The accumulation of hydrogen peroxide was determined using the method described by Jana and Choudhuri^38^ and Yusuf et al.^37^

Electrolyte leakage was assessed by measuring the total inorganic ions released from the leaves, following the protocol of Sullivan and Ross.^39^

Lipid peroxidation was estimated by quantifying malondialdehyde (MDA) equivalents, as outlined by Yusuf et al.^37^

### Gene expression analysis

Expression levels of abiotic stress–related genes (GmCAT1, GmSOD, and GmP5CS) were quantified using qRT-PCR. Leaf tissues collected for RNA extraction were immediately stored at −80 °C, and total RNA was isolated using the ISOLATE II RNA Plant Kit. cDNA was synthesized from 500 ng RNA per sample using the SensiFAST cDNA Synthesis Kit and subsequently diluted 1:4 for qRT-PCR, with 5 µl of cDNA used in a 20 µl reaction on a QuantStudio™ 5 system. Amplification was performed using the SensiFAST SYBR Lo-ROX Kit, with *GmActin* serving as the reference gene. Relative expression was calculated using the 2^–ΔΔCt^ method based on four biological replicates, following procedures detailed in our previous work.^31^ The specific primer sets used for the qRT-PCR are listed in Table 1.

**Table 1.** List of gene primers used in the qRT-PCR analysis.

Gene	Primers	References
*GmCAT1*	ACTACAAATTCTGG TGCTCCTA (F) TGCAAGCTTCTCC ACAAGA (R)	Li et al.^40^
*GmSOD*	TGGTCTCCATGGCT TCCAT (F) GCTAACGGTACCA TCATCA (R)	Li et al.^40^
*GmP5CS*	CGAACTGAGCTTGCAGAGGGGC (F) TCGCTTAGCCTCCTTGCCTCC (R)	Xu et al.^41^
*GmActin*	ATGGCTGATGGTGAAGACATTC (F) TCCATGCTCAATAGGGTACTTG (R)	Sun et al.^42^

### Statistical analysis

The data from the experiment were analyzed statistically using analysis of variance (ANOVA) with IBM SPSS Statistics for Windows, Version 19.0 (IBM Corp., Armonk, NY). The results are shown as the mean ± SE for each treatment, and mean comparisons were performed using the least significant difference (LSD) test, with a significance threshold set at *p *≤* *0.05.

## Experimental results

### EBL alleviates growth inhibition caused by salt and drought stress

**Figure 1.**
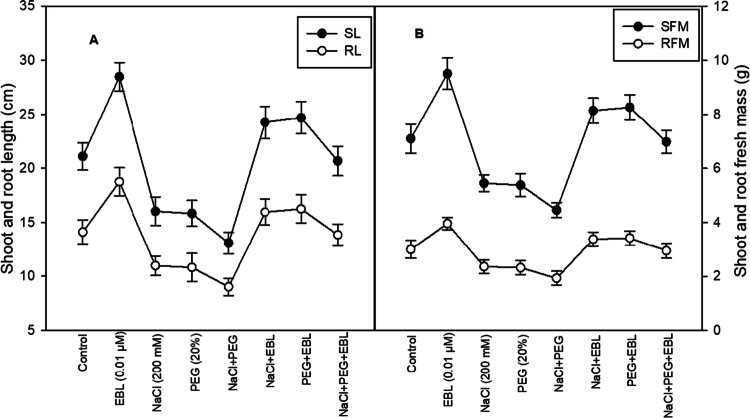
Impact of 24-epibrassinolide on (A) shoot and root length, and (B) shoot and root fresh mass of soybean plants under combined salt and drought stress at 40 d after sowing. All the data are the mean of five replicates (*n* = 5), and the vertical bars show standard errors (±SE).

Soybean growth experiences a significant decline under salt and drought stress, negatively affecting shoot length, root length, biomass, and leaf area. Salt stress reduces shoot and root length by 24% and 22.1%, respectively, while the fresh mass of shoots and roots decreases by 23.2% and 21.3% compared to control plants (Figure 1A and B). However, treatment with EBL substantially enhances shoot and root length by 35% and 33.2%, respectively, along with a 34% and 31.1% improvement in fresh mass. These results indicate that EBL effectively mitigates biomass loss due to stress, highlighting its role in promoting plant growth despite adverse environmental conditions.

### SPAD chlorophyll content

**Figure 2.**
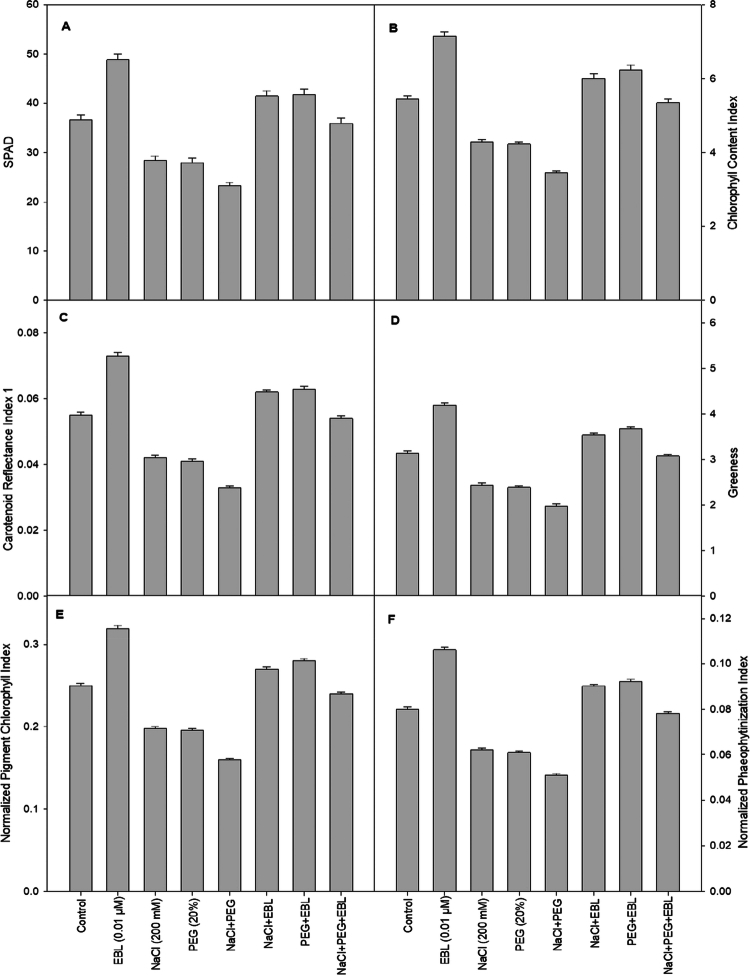
Impact of 24-epibrassinolide on (A) SPAD, (B) CCI, (C) CRI 1, (D) greenness, (E) NPCI, and (F) NPQI of soybean plants under combined salt and drought stress at 40 d after sowing. All the data are the mean of five replicates (*n* = 5), and the vertical bars show standard errors (±SE).

Salt and drought stress significantly lower SPAD chlorophyll content, with reductions of 22.4% and 23.5%, respectively, relative to that of control plants (Figure 2A). This decline suggests impaired photosynthetic efficiency and stress-induced chlorophyll degradation. However, EBL treatment leads to a 33.3% increase in SPAD values compared to untreated control plants. Under salt and drought stress, EBL-treated plants exhibit a 13.5% and 14% increase in chlorophyll content, respectively, indicating that EBL helps preserve photosynthetic pigments and enhance stress tolerance.

### Leaf spectral analysis

**Figure 3.**
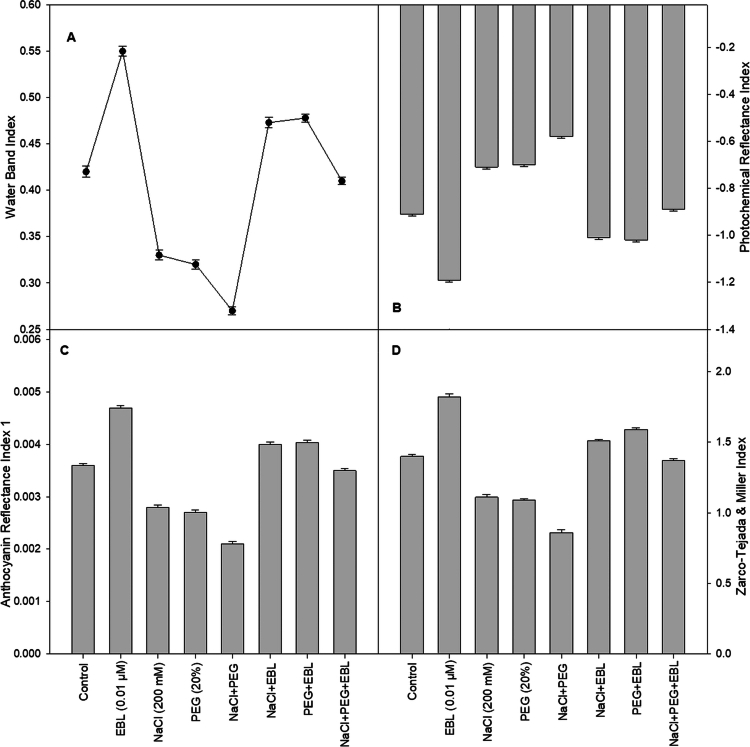
Impact of 24-epibrassinolide on (A) WBI, (B) PRI, (C) ARI 1, and (D) ZMI of soybean plants under combined salt and drought stress at 40 d after sowing. All the data are the mean of five replicates (*n* = 5), and the vertical bars show standard errors (±SE).

Spectral analysis of soybean leaves subjected to salt and drought stress reveals significant alterations in various indices. Drought-stressed plants exhibit a decline of 23.8% in ARI 1, 22% in CCI, and 24% in greenness compared to control plants, indicating reduced chlorophyll stability (Figures 2B,D, and 3C). In contrast, EBL-treated unstressed plants show improvements of 32.5% in ARI 1, 32% in CCI, and 34% in greenness, reinforcing its role in maintaining the chlorophyll content. Additionally, under salt stress, WBI, NPCI, and NPQI 1 decreased by 21.3%, 20.4%, and 22.2%, respectively (Figures 2E,F and 3A). EBL treatment enhances these indices by 32.1%, 30%, and 33.1%, respectively, suggesting improved water retention and chlorophyll stability. Moreover, a 38% reduction in ZMI is observed under combined stress conditions, whereas EBL treatment restores ZMI by 30.5%, further demonstrating its protective effects against environmental stressors (3D). Furthermore, CRI1 and PRI were reduced by NaCl, PEG, and, most markedly, by their combined application relative to the control (Figure 2C and 3B). EBL treatment elevated both indices in non-stressed plants and partly counteracted their reduction under all stress conditions.

### Rubisco activity

**Figure 4.**
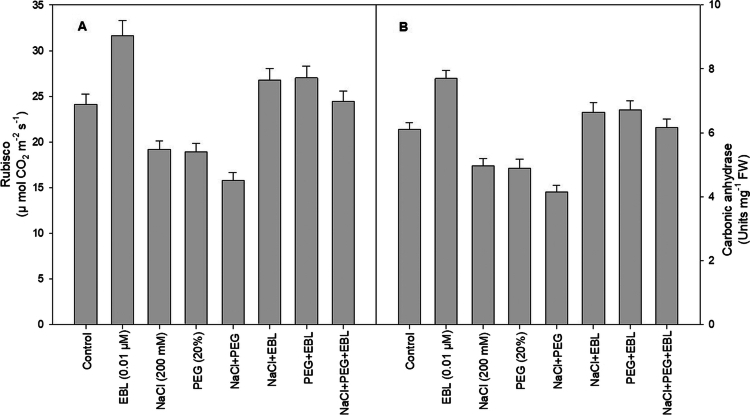
Impact of 24-epibrassinolide on (A) rubisco and (B) carbonic anhydrase of soybean plants under combined salt and drought stress at 40 d after sowing. All the data are the mean of five replicates (*n* = 5), and the vertical bars show standard errors (±SE).

Exposure to salt and drought stress significantly hampers Rubisco activity, with a combined stress scenario causing a 34.5% reduction compared to control plants. The lowest Rubisco activity is recorded under NaCl + PEG treatment. However, exogenous EBL treatment leads to a 31.2% increase in Rubisco activity compared to untreated plants (Figure 4A). When stressed plants receive EBL treatment, Rubisco activity levels improve significantly, approaching those of control plants, underscoring EBL's role in maintaining photosynthetic efficiency under adverse conditions.

### Carbonic anhydrase (CA) activity

Salt and drought stress result in an 18.4% and 19.8% reduction in CA activity, respectively, with a combined stress condition leading to a 32% decline. Foliar application of EBL increases CA activity by 27% compared to control plants (Figure 4B). Additionally, EBL treatment significantly counteracts the stress-induced decline in CA activity, promoting enhanced carbon fixation and plant metabolism.

### Photosynthetic performance under stress

**Figure 5.**
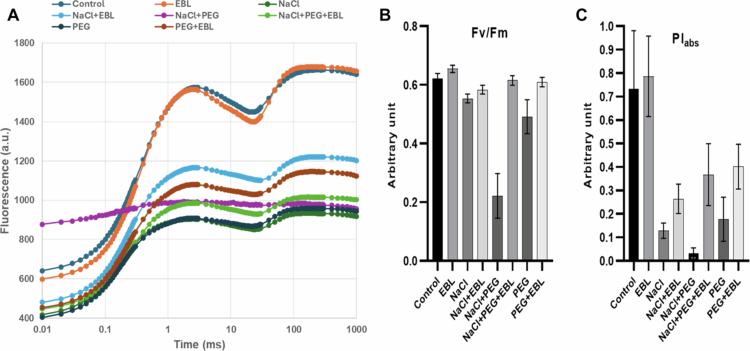
Fast chlorophyll A fluorescence kinetics in dark-adapted soybean leaves from the eight experimental groups. (A) The transient polyphasic curves for each treatment of 24-epibrassinolide in the salt- and drought-induced changes in soybean at the 40-d stage of growth, (B) quantum yield of PS II determined as Fv/Fm, and (C) performance index (PI) for each treatment of 24-epibrassinolide in the salt- and drought-induced changes in soybean at the 40-d stage of growth.

**Figure 6.**
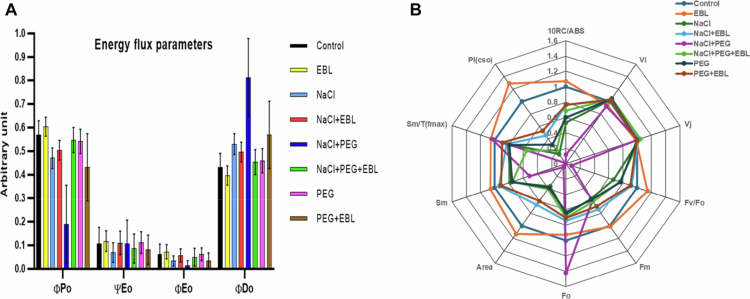
Impact of 24-epibrassinolide on (A) energy flux parameters and (B) biophysical parameters of PSII curves of soybean plants under combined salt and drought stress at 40 d after sowing.

**Figure 7.**
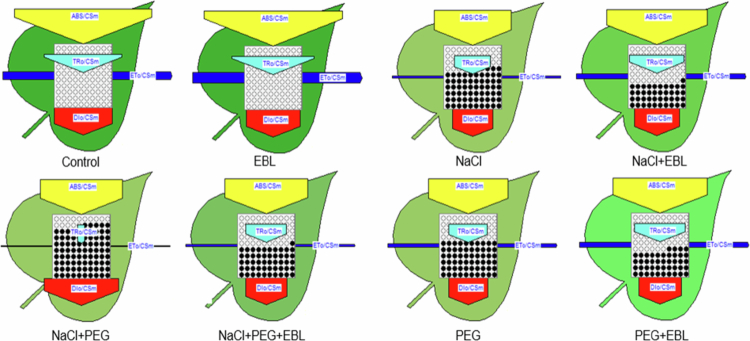
Comparative schematic showing alterations in PSII energy fluxes per cross-section (CSm) across treatments. For each treatment, absorbed photon flux (ABS/CSm), trapped energy flux (TRo/CSm), electron transport flux (ETo/CSm), and dissipated energy flux (DIo/CSm) are depicted. Increased dot density and darker leaf shading represent heightened stress, reduced quantum efficiency, and elevated non-photochemical dissipation. The diagram highlights the protective influence of 24-epibrassinolide in mitigating stress-induced declines in PSII performance under both salinity and drought stress.

A Plant Efficiency Analyzer assesses photosystem II (PSII) functionality under stress conditions. Under normal conditions, soybean plants maintain optimal PSII efficiency, with a maximum quantum yield (Fv/Fm) of approximately 0.83. However, salt (200 mM NaCl) and drought (20% PEG) stress significantly impair PSII efficiency, reducing Fv/Fm values due to photoinhibition (Figure 5B). The performance index (PI) declines sharply (Figure 5C), while non-photochemical quenching (NPQ) increases, indicating enhanced energy dissipation. Additionally, an increase in the minimum fluorescence (Fo) suggests PSII reaction center damage. Notably, EBL treatment mitigates these adverse effects, enhancing Fv/Fm values, increasing PI, and stabilizing Fo values (Figure 5). EBL-treated plants exhibit reduced NPQ, signifying improved photochemical efficiency, while energy flux parameters (ABS/RC, TRo/RC, and ETo/RC) partially recover, demonstrating EBL's role in preserving PSII stability and overall photosynthetic performance (Figures 6 and 7).

### Antioxidant enzyme activity

**Figure 8.**
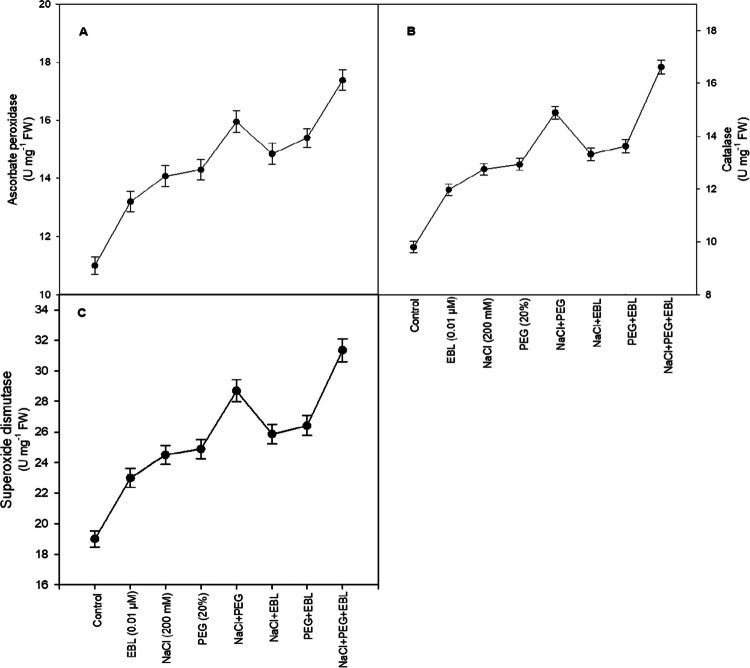
Impact of 24-epibrassinolide on (A) ascorbate peroxidase, (B) catalase, and (C) superoxide dismutase of soybean plants under combined salt and drought stress at 40 d after sowing. All the data are the mean of five replicates (*n* = 5), and the vertical bars show standard errors (±SE).

Salt and drought stress trigger significant increases in antioxidant enzyme activity, reflecting an adaptive response to oxidative stress. APX activity rises by 28% and 30%, SOD by 29% and 31%, and CAT by 30% and 32% under individual stress conditions. Under combined stress, APX, SOD, and CAT activities increase by 39%, 51%, and 52%, respectively (Figure 8A–C). EBL treatment further amplifies antioxidant responses, elevating APX, SOD, and CAT activities by 55%, 65%, and 69.6%, respectively, compared to control plants. These findings suggest that EBL fortifies the antioxidant defense system, enhancing stress resilience.

### Proline, ProDH, and P5CS activity

**Figure 9.**
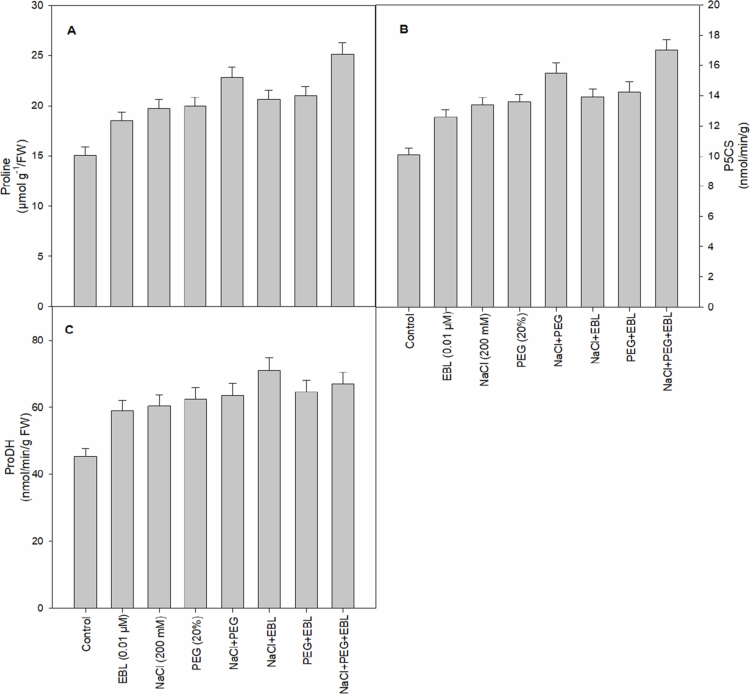
Impact of 24-epibrassinolide on (A) proline, (B) P5CS, and (C) ProDH of soybean plants under combined salt and drought stress at 40 d after sowing. All the data are the mean of five replicates (*n* = 5), and the vertical bars show standard errors (±SE).

Proline accumulation significantly increases under stress conditions, with EBL further boosting proline levels by 22.5%, highlighting its role in stress adaptation (Figure 9A). Under extreme stress, EBL treatment leads to a 66.5% rise in proline content, reinforcing its protective effects. Additionally, EBL enhances ProDH and P5CS activities by 30% and 24.7%, respectively, promoting proline synthesis and turnover (Figure 9B and C). The highest P5CS activity is observed in plants subjected to combined salt and drought stress with EBL, indicating its ability to modulate osmotic balance and mitigate oxidative stress.

### Malondialdehyde content

**Figure 10.**
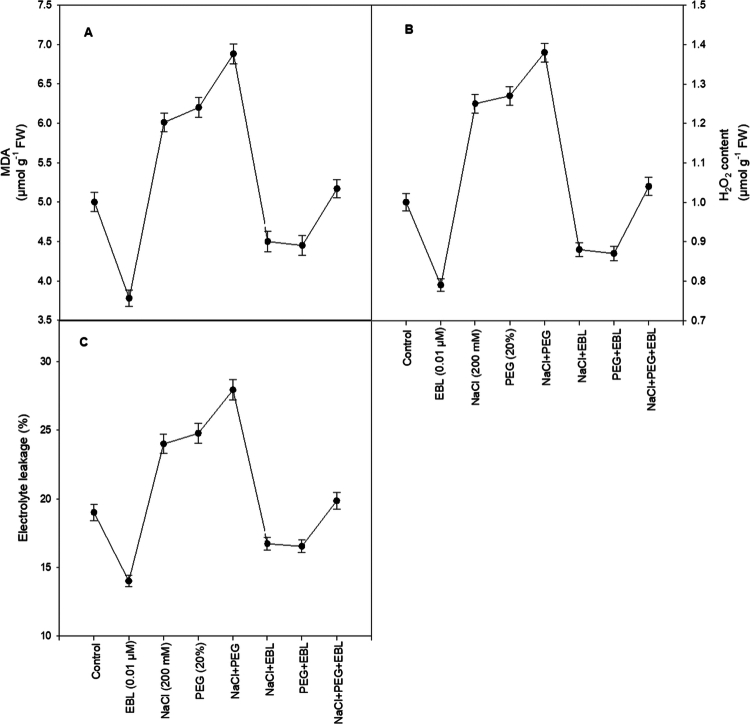
Impact of 24-epibrassinolide on (A) MDA, (B) H_2_O_2_ content, and (C) electrolyte leakage of soybean plants under combined salt and drought stress at 40 d after sowing. All the data are the mean of five replicates (*n* = 5), and the vertical bars show standard errors (±SE).

MDA levels, an indicator of lipid peroxidation, significantly increase under salt and drought stress, signaling oxidative damage. However, EBL treatment markedly reduces MDA content by 24.3%, underscoring its potential to mitigate lipid peroxidation and enhance cellular integrity (Figure 10A).

### H_2_O_2_ content

Exogenous EBL alone significantly lowered H_2_O_2_ content compared with the control, reflecting its inherent antioxidative potential (Figure 10B). Conversely, combined NaCl and PEG stress markedly elevated H_2_O_2_ accumulation. Notably, EBL co-application with NaCl, PEG, or NaCl+PEG effectively mitigated this oxidative surge, bringing H_2_O_2_ levels close to those of the control.

### Electrolyte leakage (EL)

Salt and drought stress leads to a 30% and 32% rise in EL, respectively, indicating substantial membrane damage. Combined stress further exacerbates EL, causing a 46% increase (Figure 10C). However, EBL treatment significantly reduces EL by 21%, demonstrating its protective effect on membrane stability and ion homeostasis. These results suggest that EBL strengthens cellular membranes, preventing excessive ion leakage and enhancing stress tolerance in soybean plants.

### Gene expression

**Figure 11.**
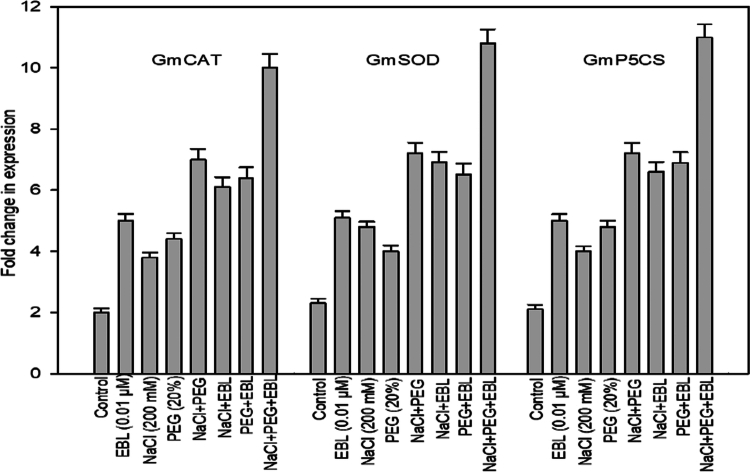
Relative expression levels of GmCAT, GmSOD, and GmP5CS genes in soybean plants under combined salt and drought stress at 40 d after sowing. The relative gene expression levels were standardized against those of Gmactin. The data represent the mean ± SE of three independent experiments, each with four biological replicates.

The expression levels of *GmCAT, GmSOD,* and *GmP5CS* were significantly influenced by NaCl, PEG, and exogenous EBL treatments, both individually and in various combinations (Figure 11). However, EBL alone resulted in a mild increase in expression, whereas individual treatments with NaCl (200 mM) and PEG (20%) triggered moderate induction, with NaCl having a comparatively stronger effect. The combined NaCl and PEG treatment further amplified gene expression, indicating an additive impact of salt and osmotic stress. A greater enhancement was observed when EBL was co-applied with either NaCl or PEG. The most pronounced upregulation of *GmCAT, GmSOD*, and *GmP5CS* was recorded under the combined treatment of NaCl, PEG, and EBL, with expression levels rising more than 10-fold compared to the control. These results demonstrate the synergistic role of EBL in promoting stress-responsive gene expression and improving plant tolerance to combined abiotic stress.

## Discussion

Abiotic stresses, particularly salinity and drought, present major challenges to agricultural productivity because they disrupt photosynthesis, impair nutrient uptake, reduce water availability, and cause oxidative damage to cellular structures, ultimately resulting in stunted growth, reduced biomass, and yield losses.^3^^,^^43–45^ In our study, salt and drought stresses markedly reduced shoot and root length and fresh and dry mass compared with those of control plants, indicating that biomass accumulation was severely compromised, most likely due to impaired cell expansion, disrupted metabolism, and reduced efficiency of water and nutrient uptake.^4^^,^^46^^,^^47^ The study reveals that EBL-treated plants show substantial improvements in shoot and root length, fresh mass, and dry weight when compared to their stressed counterparts (Figure 1A and B). These growth-promoting effects under combined salt and drought stress underscore the originality of our findings, as they demonstrate that a single hormonal treatment (EBL) can coordinately improve plant architecture, biomass accumulation, and stress resilience in soybean. These findings suggest that EBL enhances biomass accumulation and overall plant growth by promoting cellular processes such as elongation and differentiation, potentially through the activation of stress-responsive genes and pathways.^30^^,^^48^

According to Xie et al.^49^ BR has significant transcriptional effects that play a vital role in improving agricultural productivity and crop resilience. The BR signaling pathway regulates essential processes such as cell growth and division, which are key to biomass accumulation. In *A. thaliana*, BR directly influences the expression of *CELLULOSE SYNTHASE* (CESA) genes, promoting plant development by enhancing cell elongation and increasing cellulose content, leading to greater biomass.^49^ Environmental stresses like high salinity hinder cell elongation and disrupt the cell cycle by suppressing the expression of genes, including CESA and specific cyclins.^50^^,^^51^ Additionally, BR signaling upregulates a suite of cell wall-modifying enzymes such as expansins, xyloglucan endotransglucosylase/hydrolases, and pectin-degrading enzymes, which mediate cell wall loosening and restructuring. By sustaining the activity of these enzymes under stress, EBL likely preserves wall plasticity and allows continued cell expansion even under osmotic and ionic constraints.^52^^,^^53^ By linking these known BR-regulated cell wall processes with our observed improvements in shoot and root growth, our data suggest that EBL maintains the cell expansion machinery even under osmotic and ionic stress, thereby offering a mechanistic explanation for the higher biomass in treated plants. BR has also been shown to mitigate heat stress effects on cell growth.^23^^,^^54^ This crosstalk between BR signaling, CESA regulation, and tolerance to multiple stresses provides a coherent framework for interpreting our results under combined stress scenarios rather than representing an unrelated effect of heat stress. Furthermore, Khan et al.^29^ reported increases in the fresh and dry weight of wheat and pea after EBL treatment, indicating the practical benefits of BR in agriculture. Our work extends these observations to soybean and, importantly, integrates cell wall–related responses with whole-plant performance under simultaneous salt and drought stress, an interaction that has been much less explored.

Spectral analysis of soybean plants under salt and drought stress provided additional insight into their physiological responses. Drought stress led to a significant decrease in ARI1, CCI, and greenness (Figures 2 and 3), indicating a reduced chlorophyll content and photosynthetic efficiency, which could negatively affect growth and productivity.^55^^,^^56^ EBL treatment of stress-free plants improved these indices, suggesting that EBL stabilizes chlorophyll and enhances photosynthesis under normal conditions. Salt stress resulted in reduced WBI, NPCI, and NPQI compared to controls, indicating impaired water retention, disrupted pigment balance, and potential cellular damage from ionic toxicity.^57^ EBL treatment improved these indices, highlighting its role in preserving water status and pigment integrity under salinity. Similar declines in spectral indices such as NDVI, PSRI, and CRI1 under NaCl treatment have been reported in Arabidopsis.^11^ The combined salt and drought stress led to more pronounced decreases in these indices, reflecting oxidative damage to chlorophyll and impaired photosynthesis.^11^ A distinctive aspect of our study is the integration of spectral traits with biochemical and molecular responses under EBL treatment, showing that improvements in ARI1, CCI, and water-related indices are tightly associated with the enhanced antioxidant capacity and osmotic adjustment described below. This integrative approach provides a new, non-destructive framework for monitoring BR-induced stress tolerance in crops.

We also observed that heat and drought stress significantly impaired photosynthesis-related parameters, resulting in a decline in photosystem II (PSII) efficiency (Figures 5–7). The initiation of senescence, coupled with alterations in enzyme activity, can influence cytoplasmic composition and sink activity, ultimately reducing the transport of photosynthates and photosynthetic rates.^58^ Previous studies have shown that heat, salinity, and drought, individually or in combination, adversely affect transpiration and photosynthesis through stomatal and non-stomatal limitations, including reduced leaf growth, senescence, and damage to the photosynthetic apparatus.^59-62^ In such situations, limited internal CO₂ availability and the inhibition of key photosynthetic enzymes and ATP synthases exacerbate the decline in photosynthesis.^63-65^ In our experiment, EBL-treated plants exhibited enhanced photosynthetic performance under stress, with improved CO₂ adjustment and more efficient light-harvesting, as indicated by increased chlorophyll content and PSII efficiency. These outcomes are consistent with earlier studies showing that exogenous BRs improve photosynthesis and the quantum yield of PSII under adverse conditions.^20^^,^^29^^,^^37^^,^^66^ Notably, our simultaneous assessment of PSII efficiency, chlorophyll-related spectral indices, and growth responses indicates that EBL does not merely preserve photosynthesis but reshapes the whole photosynthetic apparatus to function more efficiently under combined stresses, highlighting the comprehensive nature of the BR-mediated protective response.

Plants employ sophisticated mechanisms to counteract oxidative stress caused by reactive oxygen species (ROS), which are by-products of metabolic processes that accumulate under environmental stress.^67^ Excess ROS can damage lipids, proteins, and nucleic acids, ultimately impairing plant growth and productivity. To mitigate oxidative damage, plants rely on both non-enzymatic and enzymatic antioxidant defenses.^68^ Non-enzymatic components include molecules such as proline, which help stabilize cell structures and scavenge ROS,^69^ whereas enzymatic defenses involve key antioxidant enzymes such as SOD, CAT, APX, and POX, which detoxify ROS by converting them into less harmful species.^70^ Our study revealed that EBL treatment significantly increased antioxidant enzyme levels, particularly CAT, APX, and SOD activities under drought stress (Figure 8A–C), indicating that EBL strengthens the antioxidant defense system. This enhancement is linked to the upregulation of the *DET2* gene, which is essential for brassinosteroid biosynthesis,^71^ which in turn may increase the availability of endogenous BRs that regulate antioxidant-related genes. Previous studies reported that exogenous BRs enhance antioxidant enzyme activities during abiotic stress.^66^^,^^72^ Our results add originality by directly associating *DET2* upregulation with elevated antioxidant enzyme activities and improved physiological performance under combined salt and drought stress, thereby providing a mechanistic bridge between BR biosynthesis and ROS detoxification that has rarely been demonstrated in soybean.

Proline acts as a signaling molecule that helps control mitochondrial activities and trigger gene expression, aiding plant recovery from abiotic stress.^73^ In agreement with this, our study observed the highest proline accumulation in plants treated with EBL under combined heat and drought stress (Figure 9A). These findings suggest that EBL activates multiple stress-response pathways, enhancing proline synthesis to alleviate the damaging effects of concurrent stresses.^74^^,^^75^ Proline's dual role as an osmolyte and antioxidant is essential in protecting plants from severe cellular damage and metabolic imbalances. The highest enzymatic activities of both Δ¹-pyrroline-5-carboxylate synthase (P5CS) and proline dehydrogenase (ProDH) were observed in plants exposed to combined salt and drought stress with EBL treatment, further underscore the effectiveness of EBL in managing proline metabolism under severe stress conditions (Figure 9B and C). Increased P5CS activity has previously been associated with EBL-induced proline synthesis in wheat under aluminum and salt stress,^37^ whereas elevated ProDH activity suggests that EBL ensures proline turnover, preventing potential negative effects of excessive proline accumulation, such as feedback inhibition of biosynthetic pathways. Under stress conditions, BRs are known to promote proline accumulation by upregulating genes associated with proline biosynthesis.^76^^,^^77^ By demonstrating that EBL simultaneously enhances P5CS and ProDH activities, our study provides novel evidence that BRs fine-tune both proline accumulation and its turnover, enabling dynamic osmotic adjustment rather than simple proline accumulation under multi-stress conditions. Overall, our findings demonstrate that EBL fine-tunes proline metabolism by simultaneously enhancing its biosynthesis and turnover, thereby enabling plants to maintain osmotic balance more effectively and to better mitigate oxidative damage under abiotic stress conditions.

Under various abiotic conditions, MDA levels, H₂O₂ accumulation, and electrolyte leakage (EL) act as key biochemical indicators of free-radical-induced damage.^78^ Salt and drought stresses commonly disrupt ion homeostasis and cause membrane damage, resulting in increased EL and ion toxicity. In our experiment, non-treated plants subjected to salt and drought stress showed marked increases in MDA, H₂O₂, and EL (Figure 10A–C), confirming that oxidative stress plays a central role in the injury caused by these environmental constraints.^79^^,^^80^ When considered together with the enhanced antioxidant enzymes and proline metabolism, the reductions in MDA, H₂O₂, and EL in EBL-treated plants reveal a coherent EBL-driven protective network that stabilizes membranes, maintains ion homeostasis, and prevents uncontrolled ROS increase under combined stresses. Much research indicates that abiotic stress conditions can trigger oxidative bursts, leading to an overproduction of ROS and disruption of the delicate balance between ROS production and detoxification mechanisms.^81^ While both EBL and ROS are pivotal in plant responses to stress, they exhibit distinct roles: ROS primarily function as signaling molecules but can cause cellular damage when their levels become excessive, whereas EBL acts as a protective agent, enhancing several physiological and biochemical processes to mitigate stress impacts. Taken together, our data show that EBL orchestrates a multi-layered defense, spanning cell expansion, photosynthetic performance, antioxidant machinery, and osmotic adjustment, which confers robust tolerance to complex abiotic stress combinations in soybean, highlighting its strong potential as a practical tool for climate-resilient agriculture.

## Conclusion

**Figure 12.**
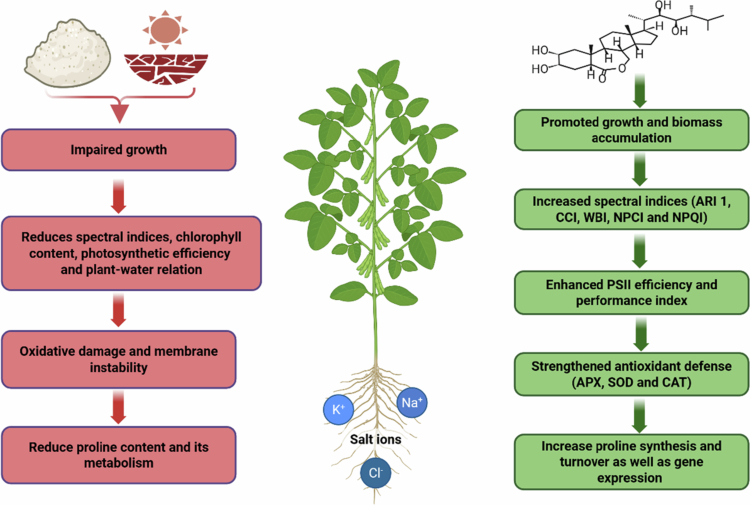
EBL as a valuable growth regulator for crop resilience to environmental stressors.

This study demonstrates that EBL effectively mitigates the adverse effects of salt and drought stress on soybean plants (Figure 12). and drought-imposed limitations on growth, photosynthetic efficiency, and membrane stability were substantially alleviated by EBL, which promoted biomass accumulation, sustained chlorophyll content, and improved water status. Increased SPAD values and enhanced spectral indices (ARI1, CCI, WBI, NPCI, and NPQI) in EBL-treated plants indicate more efficient light harvesting and better photosystem functionality under stress. The parallel improvements in PSII efficiency and the performance index further confirm reduced photoinhibition. EBL also strengthened antioxidant defense by enhancing APX, SOD, and CAT activities and stimulated proline synthesis and turnover, thereby supporting osmotic adjustment and limiting oxidative damage. The lower MDA content and reduced EL in EBL-treated plants reflect improved membrane integrity and protection against lipid peroxidation. Collectively, these findings identify EBL as a promising regulator to enhance soybean resilience to salt and drought stress.
